# Supplementary material for: Computational analysis of stochastic delay dynamics in maize streak virus
Source: PLoS One. 2025 Dec 12;20(12):e0337556. doi: 10.1371/journal.pone.0337556 (PMC12700465; doi:10.1371/journal.pone.0337556)
Supplement: Attachment — Submitted filename: Review comment.pdf [file pone.0337556.s001.pdf]

Review Report on Manuscript Number : PONE-D-25-54354 Computational and Bioinformatics Analysis of Stochastic Delay Dynamics in MaizeStreak Virus

Comments:

- **Abstract:** The current abstract does not logically present the study's findings. Please restructure it to clearly outline:
  - Objectives
  - Methodology
  - Key results
  - Conclusions

  in a coherent sequence. Make the Abstract with proper allignment.
- **Research Gap:** Add a dedicated subsection under "Research Gap" to the introduction, explaining how this study addresses limitations in existing literature.
- **Objectives and Findings:** You may summarize the key objectives and findings in key points after research gap.
- **Completeness:** Verify that all references, figures, and tables are properly cited in the text. Some citations looks not in proper format. You may revise carefully.
- Numerous grammatical and punctuation errors exist throughout. It have to be revised. You may revise specifically **Abstract**, **Introduction** and **Conclusion** section carefully.
- Revise similar errors across the entire document.
- **Section 2** In the Formulation of the model description, the model figure **(Figure 1)** should be modified for better clarity, with clearer compartment labeling and distinct transmission pathways.
- **Table** Please clarify the process or method used to calculate or estimate each parameter for better transparency and reproducibility. Summarize model parameters in a Table 1, using proper font size and allignment. Make sure all are in same structure.
- **Basic reproduction number** In Mathematical Analysis, you may include a separate section after **3.2** dedicated to the calculation of the basic reproduction number (R_0) to clearly present the derivation steps and underlying assumptions. You have to show a detailed analysis for this.
- Here are so many punctuation and grammatical errors in Definition 1 to 4. You have to correct this carefully.
- Revise all the **theorems 1 to 6** by correcting Mathematical, grammatical errors.
- Table 1 is poor structured. Revise it for well visualizations.
- A paragraph including the main summary of the study need to be modified reducing the grammatical and puncuation errors.
- **Symbols & Equations:**
  - **Table and Figures:** Make sure Symbols must be formatted as equations in the tables and figures headings. In the theorem analytical study, all mathematical symbols should be carefully revised, as some parentheses and notations appear not merged or unclear.
  - You may include a diagram reflecting sensitivity analysis of each parameters on R_0 along with analytical study for clear explanation.
- **Parameter Estimation (Table 1, 2 & Figures 2 - 9):** Parameter estimation is missing

here. The methods for parameter estimation for numerical calculation are missing. Provide detailed procedures (e.g., data sources, fitting techniques).

- **Mathematical Errors:**
  – Revise the manuscript to reduce symbolical error. As for example: use R_0 instead of $R0$.
- To focus both modeling, parameter estimation, graphs and data analysis, you may read the following articles. Update the literature-
  1. Bifurcation analysis of an influenza A (H1N1) model with treatment and vaccination. PLoS ONE 20(1): e0315280. 2025
  2. Stochastic Differential Equations to Model Influenza Transmission with Continuous and Discrete-Time Markov Chains, *Alexandria Engineering Journal*, 2025
  3. Modeling influenza transmission and control: epidemic theory insights across Mexico, Italy, and South Africa. Theory in Biosciences, 1-30.
  4. Wiener and Lévy processes to prevent disease outbreaks: Predictable vs stochastic analysis. Partial Differential Equations in Applied Mathematics 10, 100712.
- All the numerical results need to be modified for better visualization **(Figures 2 - 9)**. You may adjust line width, border, markers for the figures. For sensitivity analysis, you may add new presentation of parameters effect by heat map, phase portrait of $\mathcal{R}_0$ etc.
- You have to clarify which parameters are used to investigate the **figures 2 - 9.**
- The visulaization is not perfect. Make the figures in several rows, maximum 3 figures in each row, by enhancing size and quality, decoration.
- The Reference formats are wrong. Revise it. Make sure all the references are in same style.
- Overall the whole manuscript need to be revised very carefully.

Recommendation

The authors should rewrite the manuscript to address all noted concerns, verify all mathematical derivations and results, and resubmit only after thorough revision. Even I am not sure whether it's possible to overcome, especially the theoretical analysis. Due to the pervasive issues in clarity, mathematical correctness, and presentation, the manuscript requires substantial revision. After significance corrections and careful revision, I recommend for publication.
