# Supplementary material for: Computational analysis of stochastic delay dynamics in maize streak virus
Source: PLoS One. 2025 Dec 12;20(12):e0337556. doi: 10.1371/journal.pone.0337556 (PMC12700465; doi:10.1371/journal.pone.0337556)
Supplement: Attachment — Submitted filename: review.pdf [file pone.0337556.s002.pdf]

Review Report for the Manuscript

"Computational and Bioinformatics Analysis of Stochastic Delay Dynamics in Maize Streak Virus"

PLOS ONE – Manuscript ID: PONE-D-25-54354

Recommendation: Minor Revision

1. Technical Soundness and Data Support

Response: *Partly*
Comment: The manuscript presents a technically well-structured stochastic delay differential model for Maize Streak Virus (MSV) dynamics and evaluates multiple stochastic numerical solvers (Euler–Maruyama, Runge–Kutta, Milstein, and NSFD). The deterministic and stochastic analyses are mathematically sound, and equilibrium and stability proofs appear consistent. However, some assumptions (e.g., parameter estimation, justification for delay selection, and noise intensity choice) lack empirical or biological grounding. While the computational experiments are coherent and the results support qualitative conclusions, stronger biological validation or data fitting would enhance technical rigor.

2. Statistical Analysis Rigor

Response: *Partly*
Comment: The study mainly employs numerical and stability analyses rather than formal statistical inference. The simulations effectively illustrate stability and convergence properties, but there is limited quantitative assessment (e.g., variance analysis, convergence error metrics, or sensitivity quantification). Inclusion of parameter sensitivity or stochastic variance comparison would increase rigor and reproducibility.

3. Data Availability

Response: *Partly*
Comment: The authors state that datasets are available "upon reasonable request." According to PLOS Data Policy, this does **not** meet the requirement for full availability. Either the numerical codes and parameter tables should be deposited in a public repository (e.g., GitHub, Zenodo) or fully included in the supplementary material. Sharing simulation scripts and parameter sets would ensure transparency and replicability.

4. Clarity and English Quality

Response: *Yes*
Comment: The manuscript is written clearly and coherently. Section organization (introduction, model formulation, analysis, numerical methods, results, and conclusion) is logical, and English usage is fluent. Minor grammatical and stylistic revisions (e.g., article use and phrasing) could improve flow, but overall readability is high and the scientific tone is appropriate.

5. Review Comments to the Author

The manuscript offers a meaningful contribution by combining **deterministic and stochastic delay modeling** with computational analysis for maize viral dynamics. The comparative evaluation of stochastic solvers, particularly the NSFD scheme provides practical insights into numerical stability under stochastic perturbations.

Strengths

- Comprehensive model formulation integrating delay and stochasticity.
- Comparative study of numerical methods with stability discussion.
- Clear derivation of equilibria and the basic reproduction number (R_0).
- Well-organized figures and convincing convergence behavior.

Areas for Improvement

1. **Parameter Justification:** Provide references or biological rationale for parameter values and delay selection.
2. **Data and Code Availability:** Include numerical code and parameter sets in supplementary materials or a repository to comply with PLOS data-sharing standards.
3. **Quantitative Validation:** Include at least a simple parameter sensitivity or stochastic variance analysis to support claims about robustness and stability.
4. **Figures and Captions:** Clarify figure legends, explicitly indicating which numerical scheme corresponds to each curve.
5. **References:** Verify citation consistency and add recent works on stochastic epidemiological models for plant diseases to strengthen contextual grounding. Here are some recommendations (DOI) authors must add:

- `10.1016/j.cnsns.2025.109246` — Presents recent advances in stochastic delay modeling within nonlinear systems, directly relevant to the paper's numerical framework.
- `10.1142/S1793524525501074` — Demonstrates a comparative stochastic–deterministic analysis in epidemic modeling, supporting the methodology used for MSV dynamics.
- `10.3329/ganit.v43i1.67858` — Explores compartmental epidemic models with intervention measures, similar in structure to the proposed plant-virus framework.
- `10.1371/journal.pone.0315280` — Conducts bifurcation and control analysis of delayed infectious disease models, reinforcing the importance of delay and stability analysis in this study.

Overall, the study is methodologically solid and of potential interest to readers in mathematical biology and computational epidemiology. With modest clarifications and data-sharing improvements, it will be suitable for publication.
