# Supplementary material for: Computational analysis of stochastic delay dynamics in maize streak virus
Source: PLoS One. 2025 Dec 12;20(12):e0337556. doi: 10.1371/journal.pone.0337556 (PMC12700465; doi:10.1371/journal.pone.0337556)
Supplement: Attachment — Submitted filename: Response-to-Reviewer .pdf [file pone.0337556.s005.pdf]

Response to comments on manuscript ID

Manuscript ID: PONE-D-25-54354

Title: Computational and Bioinformatics Analysis of Stochastic Delay Dynamics in Maize Streak Virus

Ali Raza et al.

October 28, 2025

Beforehand, we would like to thank the anonymous reviewers and the editor in charge of this manuscript for all their valuable comments and criticism. We have thoroughly revised the Paper in light of those comments, and we have tried to respond to the reviewer's questions and suggestions to the best of our capabilities. For convenience, we have highlighted the corrections and the changes in the revised Paper with different colours.

We quote the comments for the sake of convenience and respond to each of them. The changes are highlighted in the Paper using RED TEXT for reviewer 1, BLUE TEXT for reviewer 2 and GREEN TEXT for reviewer 3.

Response to Reviewer # 1
The authors should rewrite the manuscript to address all noted concerns, verify all mathematical derivations and results, and resubmit only after thorough revision. Even I am not sure whether it's possible to overcome, especially the theoretical analysis. Due to the pervasive issues in clarity, mathematical correctness, and presentation, the manuscript requires substantial revision.

RESPONSE: We sincerely thank the reviewer for the valuable feedback and careful evaluation of our work. In the revised version of the manuscript, we have thoroughly updated the analysis and model formulation to explicitly include the stochastic delay differential equation (SDDE) structure throughout the theoretical and numerical sections.

COMMENT 1. Abstract: The current abstract does not logically present the study's findings. Please restructure it to clearly outline: – Objectives – Methodology – Key results – Conclusions in a coherent sequence. Make the Abstract with proper allignment.

RESPONSE: Thanks a lot. We have thoroughly revised the Abstract to ensure a clear and logical flow following the structure recommended by the reviewer. The revised Abstract now explicitly presents the study's objectives, methodology, key findings, and conclusions in a coherent sequence. This restructuring enhances the readability and scientific clarity of the manuscript.

COMMENT 2. Research Gap: Add a dedicated subsection under "Research Gap" to the introduction, explaining how this study addresses limitations in existing literature.

RESPONSE: Thanks a lot. We appreciate the reviewer's insightful suggestion. We have added a **Research Gap"** within the Introduction. This clearly outlines the limitations in previous studies on *Maize Streak Virus* (MSV) modeling and explains how our current work addresses these gaps by introducing a stochastic delay-based computational framework and a nonstandard finite difference (NSFD) numerical approach..

COMMENT 3. Objectives and Findings: You may summarize the key objectives and findings in key points after research gap.

RESPONSE: Thanks a lot. Admitted and updated.

COMMENT 4. Completeness: Verify that all references, figures, and tables are properly cited in the text. Some citations looks not in proper format. You may revise carefully

RESPONSE: Thanks a lot. Admitted and updated.

COMMENT 5. Numerous grammatical and punctuation errors exist throughout. It have to be revised. You may revise specifically Abstract, Introduction and Conclusion section carefully.

RESPONSE: Thanks a lot. Admitted and updated.

COMMENT 6. Revise similar errors across the entire document.

RESPONSE: Thanks a lot. Admitted and updated.

COMMENT 7. Section 2 In the Formulation of the model description, the model figure (Figure 1) should be modified for better clarity, with clearer compartment labeling and distinct transmission pathways.

RESPONSE: Thanks a lot. Admitted and updated.

COMMENT 8. Table Please clarify the process or method used to calculate or estimate each parameter for better transparency and reproducibility. Summarize model

parameters in a Table 1, using proper font size and allignment. Make sure all are in same structure.

RESPONSE: Thanks a lot. Admitted and updated.

COMMENT 9. Basic reproduction number In Mathematical Analysis, you may include a separate section after 3.2 dedicated to the calculation of the basic reproduction number (R0) to clearly present the derivation steps and underlying assumptions. You have to show a detailed analysis for this.

RESPONSE: Thanks a lot. Admitted and updated.

COMMENT 10. Here are so many punctuation and grammatical errors in Definition 1 to 4. You have to correct this carefully.

RESPONSE: Thanks a lot. Admitted and updated.

COMMENT 11. Revise all the theorems 1 to 6 by correcting Mathematical, grammatical errors..

RESPONSE: Thanks a lot. Admitted and updated

COMMENT 12. Table 1 is poor structured. Revise it for well visualizations.

RESPONSE: Thanks a lot. Admitted and updated.

COMMENT 13. A paragraph including the main summary of the study need to be modified reducing the grammatical and puncuation errors.

RESPONSE: Thanks a lot. Admitted and updated.

COMMENT 14. Symbols & Equations: – Table and Figures: Make sure Symbols must be formatted as equations in the tables and figures headings. In the theorem analytical study, all mathematical symbols should be carefully revised, as some parentheses and notations appear not merged or unclear. – You may include a diagram reflecting sensitivity analysis of each parameters on R0 along with analytical study for clear explanation.

RESPONSE: Thanks a lot. Admitted and updated.

COMMENT 15. Parameter Estimation (Table 1, 2 & Figures 2 - 9): Parameter estimation is missing 1 here. The methods for parameter estimation for numerical calculation are missing. Provide detailed procedures (e.g., data sources, fitting techniques).

RESPONSE: Thanks a lot. We take a parameters value from scientific literature, so we have updated a concerned table.

COMMENT 16. Mathematical Errors: – Revise the manuscript to reduce symbolical error. As for example: use R0 instead of R0.

RESPONSE: Thanks a lot. Admitted and updated.

COMMENT 17. To focus both modeling, parameter estimation, graphs and data analysis, you may read the following articles. Update the literature1. Bifurcation analysis of an influenza A (H1N1) model with treatment and vaccination. PLoS ONE 20(1): e0315280. 2025 2. Stochastic Differential Equations to Model Influenza Transmission with Continuous and Discrete-Time Markov Chains, Alexandria Engineering Journal, 2025 3. Modeling influenza transmission and control: epidemic theory insights across Mexico, Italy, and South Africa. Theory in Biosciences, 1-30. 4. Wiener and L´evy processes to prevent disease outbreaks: Predictable vs stochastic analysis. Partial Differential Equations in Applied Mathematics 10, 100712.

RESPONSE: Thanks a lot. Admitted and updated.

COMMENT 18. All the numerical results need to be modified for better visualization (Figures 2 - 9). You may adjust line width, border, markers for the figures. For sensitivity analysis, you may add new presentation of parameters effect by heat map, phase portrait of R0 etc

RESPONSE: Thanks a lot. We have added figures comparing the performance of different numerical methods. At this stage, our focus is not on sensitivity analysis. However, your suggestions are greatly appreciated, and we plan to incorporate them in our future and upcoming research papers.

COMMENT 19. You have to clarify which parameters are used to investigate the figures 2 - 9

RESPONSE: Thanks a lot. Admitted and updated..

COMMENT 20. The visulaization is not perfect. Make the figures in several rows, maximum 3 figures in each row, by enhancing size and quality, decoration

RESPONSE: Thanks a lot. Admitted and updated.

COMMENT 21. The Reference formats are wrong. Revise it. Make sure all the references are in same style.

RESPONSE: Thanks a lot. Admitted and updated.

COMMENT 22. Overall the whole manuscript need to be revised very carefully.

RESPONSE: Thanks a lot. Admitted and updated.

Recommendation The authors should rewrite the manuscript to address all noted concerns, verify all mathematical derivations and results, and resubmit only after thorough revision. Even I am not sure whether it's possible to overcome, especially the theoretical analysis. Due to the pervasive issues in clarity, mathematical correctness, and presentation, the manuscript requires substantial revision. After significance corrections and careful revision, I recommend for publication.

RESPONSE: We sincerely thank the reviewer for the valuable feedback and careful evaluation of our work.

Response to Reviewer # 2

This manuscript presents a comprehensive and mathematically rigorous analysis of a stochastic delayed differential equation model for Maize Streak Virus (MSV) dynamics. The work is well-structured, moving from a deterministic formulation to a complex stochastic model with time delays. The analytical proofs for positivity, boundedness, equilibrium stability, and disease extinction are thorough.

A significant strength of the paper is the detailed comparison of various stochastic numerical methods (Euler-Maruyama, Runge-Kutta, NSFD), with a clear demonstration of the advantages of the stochastic Non-Standard Finite Difference (NSFD) scheme in terms of stability and step-size independence. The discussion on the impact of time delay (τ) on the basic reproduction number (R_0) and disease dynamics is particularly insightful and valuable for understanding control strategies. The manuscript is suitable for publication in PLOS ONE after the authors address the following points, which primarily aim to clarify methodology and strengthen the discussion.

RESPONSE: Thanks a lot. We are grateful for the valuable comments and suggestions provided by the anonymous referee.

COMMENT 1. Model Parameterization and Validation: The parameters in Table 2 are listed as "Fitted" or "Estimated." Could you please provide more detail on the data source and the procedure used for this parameter estimation or fitting? Furthermore, how were the values for the stochastic noise intensities (σ_1 to σ_5) chosen for the simulations in Section

RESPONSE: Thanks a lot. We have taken the parameter values from the scientific literature, and the corresponding sources have been cited. The primary focus of the present study is on the computational analysis and the efficiency of the proposed numerical methods for the given data.

COMMENT 2. Clarification of Stochastic Formulations: The manuscript presents two different stochastic formulations (Phase 1 in Eqs. 9-10 and Phase 2 in Eqs. 11-15). Phase 1 uses a complex diffusion matrix derived from transition probabilities, while Phase 2 uses a simpler, more common linear multiplicative noise. Could you clarify the relationship between these two approaches and justify why the analysis and numerical simulations primarily focus on the model from Phase 2?

RESPONSE: Thanks a lot. The two stochastic formulations are conceptually connected but serve different purposes in the model development process. **Phase 1** represents the *theoretical derivation* of the stochastic system based on transition probabilities and diffusion approximations of the discrete events governing infection, recovery, and treatment processes. This phase establishes the general stochastic framework and defines how random fluctuations enter the system dynamics.

Phase 2, on the other hand, provides a *simplified but equivalent representation* of the same system using linear multiplicative noise terms associated with each compartment. This formulation is mathematically tractable, allows the use of Itô calculus for analytical proofs (e.g., positivity, boundedness, and extinction), and is more practical for implementing numerical schemes such as the stochastic NSFD, Euler–Maruyama, and Runge–Kutta methods.

Therefore, the analysis and simulations focus on **Phase 2**, as it preserves the essential stochastic properties derived in Phase 1 while enabling rigorous stability analysis and efficient numerical computation.

COMMENT 3. "Bioinformatics" in the Title and Scope: The term "Bioinformatics" is prominently featured in the title, yet the manuscript is predominantly a mathematical and computational modeling study. Could you elaborate on the specific bioinformatics analyses performed (e.g., sequence data analysis, phylogenetic analysis) or clarify the usage of the term to align better with the paper's core content, perhaps focusing on "computational biology"?

RESPONSE: Thanks a lot. We appreciate the reviewer's observation and agree that the manuscript primarily focuses on mathematical and computational modeling rather than direct bioinformatics analyses such as sequence alignment or phylogenetic reconstruction. In the earlier draft, the term "Bioinformatics" was used in a broader sense to reflect the *computational analysis of biological systems* through mathematical frameworks.

To better align the title and scope with the study's actual focus, we have revised the terminology to emphasize **computational biology and stochastic modeling** rather than bioinformatics. Accordingly, the title has been updated to:

"Computational Analysis of Stochastic Delay Dynamics in Maize Streak Virus"

This modification ensures the title accurately represents the paper's content, which integrates computational and stochastic techniques to understand plant viral dynamics.

COMMENT 4. Numerical Comparison Metrics: The conclusion states that the stochastic NSFD approach is the "most accurate, productive, and efficient." To strengthen this claim, could you provide quantitative metrics for this comparison, such as computational time (CPU time), mean squared error compared to a benchmark solution, or stability region analyses for the different methods?

RESPONSE: Thanks a lot. we have incorporated quantitative performance metrics comparing the stochastic NSFD, Euler–Maruyama, and stochastic Runge–Kutta methods. These metrics now include:

- Computational efficiency: Average CPU time (in seconds) for 1,000 simulation runs.
- Numerical accuracy: Mean squared error (MSE) of each method relative to a deterministic benchmark solution.
- Stability analysis: The maximum permissible time step (h) for which numerical stability and positivity are maintained.

The results are now summarized in Table 3 and demonstrate that the stochastic NSFD method achieves:

- Approximately 35–40% lower CPU time,
- 50–60% smaller MSE, and
- Step-size–independent stability, whereas the Euler–Maruyama and Runge–Kutta methods become unstable for $h>0.5$.

These quantitative comparisons substantiate our conclusion that the stochastic NSFD method is the most stable, accurate, and computationally efficient scheme among those tested.

COMMENT 5. Biological Interpretation of Delay (τ): The time delay τ is a critical component of the model. While its mathematical impact is well-demonstrated, could you provide a more detailed biological interpretation? What specific biological processes (e.g., the latent period in the insect vector, the incubation period within the plant, or a delay in implementing control measures) is this delay intended to represent?

RESPONSE: Thanks a lot. The time delay (τ) in our model represents the biological latency and transmission lag occurring in the maize streak disease cycle. Specifically, τ captures:

1. The latent or incubation period within the infected maize plant, during which the *Maize Streak Virus* (MSV) replicates inside host tissues before visible symptoms appear or the plant becomes infectious.
2. The latency period in the leafhopper vector, reflecting the time between the insect acquiring the virus from an infected plant and its ability to transmit it to healthy plants.
3. Possible delays in implementing control measures, such as insecticide application or plant removal after infection detection.

Incorporating τ thus allows the model to account for these biological and operational delays, which significantly influence the timing and intensity of epidemic outbreaks. Biologically, increasing τ corresponds to prolonged viral incubation or delayed control responses, both of which elevate the risk of widespread infection a behavior consistent with our numerical results (Figures 8–10), where longer delays lead to higher infection prevalence and greater instability.

COMMENT 6. Impact of Insecticide Treatment: The parameter γ represents the efficacy of the insecticide treatment. The analysis shows its role in the reproduction number R_0, but could you discuss the simulation results that specifically illustrate the impact of varying γ on the infected plant population? This would provide direct insights into the effectiveness of insecticide-based control strategies.

RESPONSE: Thanks a lot. The influence of insecticide efficacy (γ) on disease transmission is inherently reflected in both the analytical expression of R_0 and the simulation outputs. As γ increases, the term $(1-\gamma)\beta F e^{-\mu\tau}$ in the infection rate decreases, leading to a reduction in the number of new infections originating from the insecticide-treated class.

In our simulations, this manifests as a **progressive decline in the infected plant population** $I(t)$ and a **delayed infection peak** as γ approaches higher values (≥0.9). In contrast, lower efficacy values ($\gamma < 0.7$) correspond to higher and earlier infection peaks, consistent with less effective control. Thus, the simulations confirm that improved insecticide efficacy significantly suppresses MSV spread and enhances long-term disease control, aligning with the analytical finding that R_0 decreases monotonically with γ.

COMMENT 7. Suggested References for Inclusion

The authors are also requested to consider citing the following recent papers, which demonstrate advanced numerical techniques for solving fractional and stochastic differential equations. These references would provide a broader context for the

computational methods used in this manuscript and highlight the applicability of such schemes in complex modeling scenarios.

For advanced collocation methods:

1. Raza, A., et al. "Collocation method with Morgan-Voyce polynomials to solve the time fractional long memory Black-Scholes model with jump process." Scientific Reports (2024). This work showcases a high-precision numerical technique for financial models with memory and jumps, which is methodologically relevant to the stochastic schemes discussed here.

For enhanced numerical solutions of fractional dynamics:

2. Raza, A., et al. "Enhanced numerical solution for time fractional Kuramoto–Sivashinsky dynamics via shifted companion Morgan–Voyce polynomials." Scientific Reports (2024). This paper presents an efficient spectral method for nonlinear fractional PDEs, underscoring the importance of robust numerical solvers in computational biology and other fields.

RESPONSE: Thanks a lot. We have acknowledged and cited this important work in the revised manuscript.

Response to Reviewer # 3

1. Technical Soundness and Data Support

Response: Partly

Comment: The manuscript presents a technically well-structured stochastic delay differential model for Maize Streak Virus (MSV) dynamics and evaluates multiple stochastic numerical solvers (Euler–Maruyama, Runge–Kutta, Milstein, and NSFD). The deterministic and stochastic analyses are mathematically sound, and equilibrium and stability proofs appear consistent. However, some assumptions (e.g., parameter estimation, justification for delay selection, and noise intensity choice) lack empirical or biological grounding. While the computational experiments are coherent and the results support qualitative conclusions, stronger biological validation or data fitting would enhance technical rigor.

2. Statistical Analysis Rigor

Response: Partly

Comment: The study mainly employs numerical and stability analyses rather than formal statistical inference. The simulations effectively illustrate stability and convergence properties, but there is limited quantitative assessment (e.g., variance analysis, convergence error metrics, or sensitivity quantification). Inclusion of parameter sensitivity or stochastic variance comparison would increase rigor and reproducibility.

3. Data Availability

Response: Partly

Comment: The authors state that datasets are available “upon reasonable request.” According to PLOS Data Policy, this does not meet the requirement for full availability. Either the numerical codes and parameter tables should be deposited in a public

repository (e.g., GitHub, Zenodo) or fully included in the supplementary material. Sharing simulation scripts and parameter sets would ensure transparency and replicability.

4. Clarity and English Quality

Response: Yes

Comment: The manuscript is written clearly and coherently. Section organization (introduction, model formulation, analysis, numerical methods, results, and conclusion) is logical, and English usage is fluent. Minor grammatical and stylistic revisions (e.g., article use and phrasing) could improve flow, but overall readability is high and the scientific tone is appropriate.

5. Review Comments to the Author

The manuscript offers a meaningful contribution by combining deterministic and stochastic

delay modeling with computational analysis for maize viral dynamics. The comparative evaluation of stochastic solvers, particularly the NSFD scheme provides practical insights into numerical stability under stochastic perturbations.

Strengths

- Comprehensive model formulation integrating delay and stochasticity.
- Comparative study of numerical methods with stability discussion.
- Clear derivation of equilibria and the basic reproduction number (R0).
- Well-organized figures and convincing convergence behavior.

RESPONSE: The authors would like to express their sincere gratitude to the anonymous referee for the valuable comments, constructive suggestions, and encouraging feedback.

COMMENT 1: Parameter Justification: Provide references or biological rationale for parameter values and delay selection.

RESPONSE: Thanks a lot. Table 2 has been revised to include the updated parameter values, along with appropriate citations for the sources from which these values were obtained.

COMMENT 3. Quantitative Validation: Include at least a simple parameter sensitivity or stochastic variance analysis to support claims about robustness and stability.

RESPONSE: Thanks a lot. Our primary focus in this study is the computational analysis of the proposed stochastic delay model for maize streak disease. Accordingly, we have established key mathematical properties positivity, boundedness, and dynamical consistency to demonstrate the model's robustness and validity. We sincerely appreciate the reviewer's valuable suggestion regarding additional quantitative validation. Due to current scope and data limitations, a detailed global sensitivity analysis will be explored in our future work. However, a stochastic variance analysis was conducted, showing that even under ±10% perturbations of key parameters, the

stochastic NSFD scheme maintains bounded trajectories and stable equilibria. These findings further confirm the robustness and numerical stability of the stochastic NSFD method and support the reliability of the model under parameter uncertainty.

COMMENT 4. Figures and Captions: Clarify figure legends, explicitly indicating which numerical scheme corresponds to each curve.

RESPONSE: Thanks a lot. Admitted and updated.

COMMENT 5. References: Verify citation consistency and add recent works on stochastic epidemiological
models for plant diseases to strengthen contextual grounding. Here
are some recommendations (DOI) authors must add:
2
• 10.1016/j.cnsns.2025.109246 — Presents recent advances in stochastic delay modeling within nonlinear systems, directly relevant to the paper's numerical framework.
• 10.1142/S1793524525501074—Demonstrates a comparative stochastic–deterministic analysis in epidemic modeling, supporting the methodology used for MSV dynamics.
• 10.3329/ganit.v43i1.67858 — Explores compartmental epidemic models with intervention measures, similar in structure to the proposed plant-virus framework.
• 10.1371/journal.pone.0315280 — Conducts bifurcation and control analysis of delayed infectious disease models, reinforcing the importance of delay and stability analysis in this study.

RESPONSE: Thanks a lot. We have included an important study in the literature to strengthen the paper.

COMMENT 6. Section 4, please check the issue with the delay – as stated before, as far as I can see, the delay was completely neglected here for the analysis

RESPONSE: Thanks a lot. The time delay (τ) was indeed incorporated in the model formulation and plays a role in determining the transmission dynamics through the exponential decay term $e^{-\mu\tau}$, which appears explicitly in the infection terms and in the expression of the basic reproduction number R_0.

However, in the **local stability analysis** of Section 4, the delay term was treated as a **constant exponential factor** rather than as a dynamic state variable. This approach follows standard delay differential equation analysis methods, where $e^{-\mu\tau}$ scales the

infection rate but does not alter the sign of the real parts of the eigenvalues when the delay is relatively small.

We have clarified this point in the revised manuscript by explicitly stating that **the delay was analytically accounted for through the term** $e^{-\mu\tau}$ in the Jacobian and characteristic equation, but its higher-order temporal effects were not expanded to simplify the stability derivation. This clarification ensures consistency between the model formulation and the analytical treatment presented in Section 4.
