# Supplementary material for: Computational analysis of stochastic delay dynamics in maize streak virus
Source: PLoS One. 2025 Dec 12;20(12):e0337556. doi: 10.1371/journal.pone.0337556 (PMC12700465; doi:10.1371/journal.pone.0337556)
Supplement: Attachment — Submitted filename: R2_Review comment.pdf [file pone.0337556.s004.pdf]

Review Report on Manuscript Number : PONE-D-25-54354R1 Computational Analysis of Stochastic Delay Dynamics in Maize Streak Virus.

Recommendation

After a thorough evaluation of the revised manuscript, I am satisfied that the authors have carefully and comprehensively addressed all the comments and suggestions raised during the initial round of review. The revisions have significantly improved the clarity, organization, and scientific rigor of the manuscript.
All methodological issues have been appropriately clarified, the presentation of results has been enhanced, and the discussion has been strengthened to reflect the study's contributions more effectively. The manuscript now demonstrates coherence, proper referencing, and adherence to the journal's formatting and academic standards.
Given that the authors have fulfilled all the review criteria and the manuscript now meets the required quality for scholarly publication, I am pleased to recommend it for publication.
